# 1D-3D hybrid modeling—*from multi-compartment models to full resolution models in space and time*

**DOI:** 10.3389/fninf.2014.00068

**Published:** 2014-07-29

**Authors:** Stephan Grein, Martin Stepniewski, Sebastian Reiter, Markus M. Knodel, Gillian Queisser

**Affiliations:** ^1^Computational Neuroscience, Goethe Center for Scientific Computing, Computer Science and Mathematics, Goethe UniversityFrankfurt am Main, Germany; ^2^Simulation and Modelling, Goethe Center for Scientific Computing, Computer Science and Mathematics, Goethe UniversityFrankfurt am Main, Germany

**Keywords:** calcium dynamics, electrical stimulation, hybrid, parallel, detailed modeling, intracellular signaling, neuron, PDEs

## Abstract

Investigation of cellular and network dynamics in the brain by means of modeling and simulation has evolved into a highly interdisciplinary field, that uses sophisticated modeling and simulation approaches to understand distinct areas of brain function. Depending on the underlying complexity, these models vary in their level of detail, in order to cope with the attached computational cost. Hence for large network simulations, single neurons are typically reduced to time-dependent signal processors, dismissing the spatial aspect of each cell. For single cell or networks with relatively small numbers of neurons, general purpose simulators allow for space and time-dependent simulations of electrical signal processing, based on the cable equation theory. An emerging field in Computational Neuroscience encompasses a new level of detail by incorporating the full three-dimensional morphology of cells and organelles into three-dimensional, space and time-dependent, simulations. While every approach has its advantages and limitations, such as computational cost, integrated and methods-spanning simulation approaches, depending on the network size could establish new ways to investigate the brain. In this paper we present a hybrid simulation approach, that makes use of reduced 1D-models using e.g., the NEURON simulator—which couples to fully resolved models for simulating cellular and sub-cellular dynamics, including the detailed three-dimensional morphology of neurons and organelles. In order to couple 1D- and 3D-simulations, we present a geometry-, membrane potential- and intracellular concentration mapping framework, with which graph- based morphologies, e.g., in the swc- or hoc-format, are mapped to full surface and volume representations of the neuron and computational data from 1D-simulations can be used as boundary conditions for full 3D simulations and vice versa. Thus, established models and data, based on general purpose 1D-simulators, can be directly coupled to the emerging field of fully resolved, highly detailed 3D-modeling approaches. We present the developed general framework for 1D/3D hybrid modeling and apply it to investigate electrically active neurons and their intracellular spatio-temporal calcium dynamics.

## 1. Introduction

The level of detail, that can be achieved with experimental techniques in Neuroscience is ever increasing. Intracellular organelles, protein positioning, and messenger dynamics can be recorded in spatial and temporal sequences, (Spacek and Harris, 1997; Takahashi et al., 1999; Arellano et al., 2007; Chen et al., 2008; Popov et al., 2011; Burette et al., 2012). It is obvious that processes in neurons and the brain take place in three-dimensional space and that the three-dimensionality is employed by the biological system to regulate and differentiate highly complex signaling pathways in the brain. To address the role of time-dependent, three-dimensional signal processing and investigate the role of morphology on signaling from a computational standpoint, an inevitable step is to model critical processes in the brain in three space dimensions and in time.

State of the art tools in Computational Neuroscience reduce three-dimensional problems to one-dimensional ones. For instance, simulators for the electrical signaling in neurons, e.g., NEURON (Hines and Carnevale, 2003) or Genesis (Bower and Beeman, 1997), solve a one-dimensional numerical problem in space. While this has great advantages in many applications, foremost the computational speed of the methods that allows the simulation of large network activity, the drawback is the loss of modeling the intra- and extra-cellular space of neurons, and being able to include intracellular processes in a full three-dimensional resolution. The three-dimensional organization of neurons, e.g., the filigreed geometry of the endoplasmic reticulum, (Spacek and Harris, 1997) or the intra- and inter-cellular organization of spines, (Murase and Schuman, 1999; Arellano et al., 2007; Chen et al., 2008; Tai et al., 2008; Popov et al., 2011), demonstrates the necessity for modeling intracellular processes in a highly detailed fashion in order to capture the underlying physical concepts of cellular signaling.

The need for coupling different aspects of signal processing in neurons has been recognized in the past (Kerr et al., 2008; Wils and De Shutter, 2009; Andrews et al., 2010; Cannon et al., 2010; Oliveira et al., 2010). In addition to projects focussing on the coupling of existing simulators for parallel computing architectures (Djurfeldt et al., 2010), the influence of spatial channel distribution on the electrical properties (Cannon et al., 2010) or integrating reduced intra-cellular approximations of reaction-diffusion processes, (Resasco et al., 2012; Schutter, 2013; McDougal et al., 2013a), we focus on the topic of how the three-dimensional intracellular architecture of neurons influences intra-cellular signals and how the resulting models can be efficiently solved on different computing scales. Thus, the key factors are to incorporate an accurate morphology of the neuron including the three-dimensional intra-cellular architecture (which can include active and passive organelles), model a multi-ion system described by systems of partial and ordinary differential equations (these can include the exchange mechanisms across plasma- and organelle membranes) and allow bi-directional coupling between one-dimensional membrane potential and three-dimensional intra-cellular signaling computations. The complexity of three-dimensional, detailed simulations of multi-ion systems is efficiently handled by the simulation framework uG, where Finite Element or Finite Volume discretizations and multi-grid methods are used to solve the underlying large linear equation systems on, potentially, massively parallel systems, (Heppner et al., 2013; Vogel et al., in press). Making use of uG, we propose a method to couple classical 1D-simulators for the computation of membrane potentials, with detailed 3D-simulations within an on-line 1D/3D-hybrid simulation framework.

In this paper we demonstrate this newly developed approach using the example of intra-cellular calcium dynamics coupled to the membrane potential via voltage-gated calcium channels. While 1D simulations can be carried out with classical simulators, such as NEURON or Genesis, the simulations in 3D are based on systems of partial and ordinary differential equations (PDEs and ODEs) describing distinct intracellular processes. In order to merge the two into a 1D/3D-hybrid system, two things need to be done. In the first step one needs to generate from a 1D morphology (a compartment model geometry) a surface and volume grid for numerical simulations in 3D and in the second step ensure a bi-directional coupling where the membrane potentials of the 1D simulation are mapped onto the surface of the 3D problem as boundary conditions for the numerical problem and the computed intra-cellular ion concentrations are mapped back to compute updated membrane potentials. For this we developed automated tools to compute the necessary morphology representations of a neuron and to allow bi-directional coupling.

For the neuron surface meshing in 3D, we employ a triangulation algorithm that makes use of the coordinates and radii of anatomical reconstructions, stored in e.g., hoc- or swc-format, to compute an equivalent surface mesh of the reconstructed neuron. We used TetGen (Si, 2009) to generate, from the surface grid, an intra- and extra-cellular tetrahedral volume grid. In order to associate grid nodes from the triangular surface grid with nodes from the compartment model geometry we developed *V_m_*2*uG*, a tool that uses a nearest neighbor-algorithm and kd-search algorithm to perform the mapping of membrane potential data onto the triangulated surface of the 3D-neuron.

In this paper we chose a setup consisting of a multi-compartment model of a CA1 stratum radiatum interneuron taken from Katona et al. (2011) and carried out simulations for electrical signal processing in NEURON. We then used the presented methods to couple 1D and 3D models and simulated calcium dynamics in the full three-dimensional intracellular space under various parameter settings. Based on these examples, we introduce our methods and tools for 1D/3D-hybrid modeling and show how existing models and data can be incorporated into highly detailed, three-dimensional simulations.

## 2. Results

The methods described here couple one-dimensional electrical models and three-dimensional models for intra-cellular signaling. We chose the CA1 interneuron from Katona et al. (2011) and its neuron morphology made available on *NeuroMorpho.org* (Ascoli, 2006) and on *ModelDB* (Migliore et al., 2003). Simulations of the membrane potential dynamics in 1D, i.e., on a compartment model level, were performed with NEURON (Hines and Carnevale, 2003; Carnevale and Hines, 2006), using a standard set-up defined in the Materials and Methods, section. Since intra-cellular processes are strongly regulated by calcium, e.g., (Milner et al., 1998; Bading, 1998; West et al., 2002; Clapham, 2007; Tai et al., 2008), we chose calcium dynamics regulated by plasma membrane-located calcium channels with a given density, thus modeling effectively a channel conductance density, and a diffusion-reaction process in the neuronal cytosol as a representative of three-dimensional, intracellular signaling in neurons. 3D simulations were carried out in uG, Bastian et al. (1997); Vogel et al. (in press). Note, that this is a representative setup which is applicable to any other 1D simulations and 3D intracellular processes. The coupling of both models here occurs on the level of calcium channels—these require the membrane potential in space and time on the plasma membrane, the local intra- and extra-cellular calcium concentrations, as well as the geometry itself. In this section we will introduce the models and the simulation set-up, methods for grid generation and membrane potential mapping, and will show simulation results for the described 1D/3D hybrid simulation approach.

### 2.1. The 3D calcium model

For this study we consider a calcium model on the continuum scale, including the following components:

1. **Morphology**: The morphology and thus the computational domain is defined by a standard compartment model, e.g., in the hoc-format (see Supplemental Figure S1 for an example). This morphology is then mapped to an equivalent three-dimensional computational domain.
2. **Membrane potential**: The membrane potential is an input parameter for the calcium channel models and is computed by the 1D simulations and provided to the calcium channel models as input data.
3. **Calcium channels on the plasma membrane**: Based on the models by Borg-Graham (Graham, 1999), we define N-/L- or T-type calcium channels (see Materials and Methods). Channel densities can be space-dependent, thus inhomogeneous channel distribution is possible.
4. **Cytosolic calcium dynamics**: In this study we consider diffusion of calcium and reaction of calcium with buffers in the cytosol. The computed calcium concentrations are mapped to the 1D model to compute calcium-dependent currents.

We can formulate the above points mathematically as an initial-value boundary problem for a diffusion-reaction model. To this end, let us denote the neuron geometry as Ω, which is a compact subset of ℝ^3^, such that

- Ω ⊂ ℝ^3^ with plasma membrane boundary Γ = ∂Ω,
- Ω : = Ω ∪ ∂Ω and
- Ω ∩ ∂Ω = ∅

which defines the space-time cylinder

(1) $$Z:=\Omega \times ]0,T[\subset {\mathbb{R}}^{3}\times {\mathbb{R}}^{+},\forall (x,t)\in Z:T>t>0.$$

Ω is the problem-associated domain for the 3D model, reconstructed from the original compartmental model geometry defined, in our case, by a hoc file. Furthermore let *c*($\vec{x}$, *t*) be the calcium concentration function. Then the diffusion-reaction problem can be stated as

(2) $$\frac{\partial c(\vec{x},t)}{\partial t}=D\cdot \Delta c(\vec{x},t)+R(c)\text{ in }\Omega$$

(3) $$\begin{array}{cc} \frac{\partial c(\vec{x},t)}{\partial t}=D\cdot \Delta c(\vec{x},t)+R(c)\text{ in }\Omega & \left(2\right) \end{array}$$

(4) $$\frac{\partial c(\vec{x},t)}{\partial \vec{n}}=\Phi (V_{m}(\vec{x},t),\vec{x},t),\text{ on }\partial \Omega ,$$

where *D* denotes the diffusion coefficient for cytosolic calcium, $\Delta :=\sum_{i=1}^{3}\partial^{2}/\partial x_{i}^{2}$ is the Laplace-Operator and *R*(*c*) a reaction term that depends on the calcium concentration *c*. Note that, the vector $\vec{n}$ denotes the direction perpendicular to the boundary surface. Equation (3) is the initial condition, i.e., a calcium distribution for *t* = 0 in the cytosol defined by the function *c*_0_($\vec{x}$). Function Φ defines a Neumann flux boundary condition, regulating the calcium flux through N-/L- or T-type calcium channels, and thus also depends on the space- and time-dependent membrane potential *V_m_*($\vec{x}$, *t*). Φ in this study is specified by Borg-Graham model type calcium channels, which are listed in the Materials and Methods section.

Cytosolic interaction of calcium with mobile and stationary buffers is governed by reaction equations of the type:

(5) $$B:=k_{on,B_{mobile}}[Ca^{2+}]\cdot [B_{mobile}]-k_{off,B_{mobile}}\cdot [CaB_{mobile}]$$

as well as the conservation law for the buffer concentration:

(6) $$\left[CaB_{mobile}]=B_{mobile,total}-[B_{mobile}\right]$$

The spatio-temporal dynamics of buffering molecules are described by a diffusion-process

(7) $$\frac{\partial B_{mobile}(\vec{x})}{\partial t}=D_{B_{mobile}}\cdot \Delta B_{mobile}(\vec{x})-B$$

For stationary buffers the diffusion coefficient *D_B_mobile__* can be set to zero. The parameter values of the model equations used throughout the paper are listed in Table 1.

**Table 1:** **Parameters used for the simulations using the 1D/3D hybrid framework**.

	**NEURON**	**uG**
dt [ms]	0.1	0.1
stim.dur [ms]	10	–
stim.amp [nA]	0.1	–
timesteps [#]	10000	10000
Boundary Condition	–	*V_m_* from NEURON
Calcium diffusion coefficient [μm^2^ · s^−1^]	20–100	20–100
VGCC density [μm^−2^]	200–1000	200–1000
Buffer *k_on_* [μM^−1^ · ms^−1^]	−	0.09
Buffer *k_off_* [ms^−1^]	–	0.24
Buffer diffusion coefficient [μm^2^ · ms^−1^]	–	0.043
Total buffer concentration [μM]	–	20
Extracellular calcium concentration [mM]	1.6	1.6
Intracellular calcium concentration [μM]	0.1	0.1
Temperature [K]	300	300

*The calcium diffusion coefficient was varied across the ranges documented in Allbritton et al. (1992) and the VGCC density was varied according to Pumplin et al. (1981); Eggermann et al. (2011). Values for the buffer kinetics and diffusivity were taken from Nagerl et al. (2000) as well as extracellular calcium concentration from Egelman and Montague (1999) repsectively the intra-cellular calcium concentration from Helmchen et al. (1996); Maravall et al. (2000)*.

Note, that our framework is not limited to the above example of calcium/buffer dynamics. The employed multi-physics platform uG has been successfully used in a wide variety of applications, ranging from, but not limited to, simulations of skin permeability in pharmaceutical applications (Hansen et al., 2008; Nägel et al., 2008, 2009; Muha et al., 2011) to groundwater flow studies (Grillo et al., 2010) and biogas reactor modeling (Muha et al., 2012). The latter demonstrates the use of the framework for large chemical reaction systems. The 3D intra-cellular model presented here can thus be extended to include multiple ion species that are non-linearly coupled, resulting in a non-linear system of PDEs.

### 2.2. Components of the hybrid framework

As mentioned earlier, our hybrid 1D/3D framework consists of geometry matching and the coupling of 1D membrane potential simulations and 3D intra-cellular simulations. Computational grids for numerical simulations in 3D rely on a geometry-defining surface grid and a discrete representation of the computational domain, i.e., a volume grid. Approximation of unstructured domains is ideally done by a triangular surface and tetrahedral volume grid. These grids need to be generated using the compartment geometry information, provided by the 1D model as, e.g., a hoc- or swc-file. To provide an automated way for generating surface and volume grids from anatomically reconstructed geometries, we developed novel tools and combined them with existing ones, which

1. Integrate NEURON to perform 1D simulations of membrane activity in neurons.
2. Generate triangular surface grids from hoc-style neuron morphology files.
3. Map 1D simulation data of membrane potentials from compartment geometries onto the two-dimensional neuron membrane (*V_m_*2*uG*) which are used as boundary conditions for membrane potential-dependent channels in the 3D model.
4. Integrate ion concentrations on the equivalent surface grid to feedback into the NEURON simulation.

**Figure 1:**
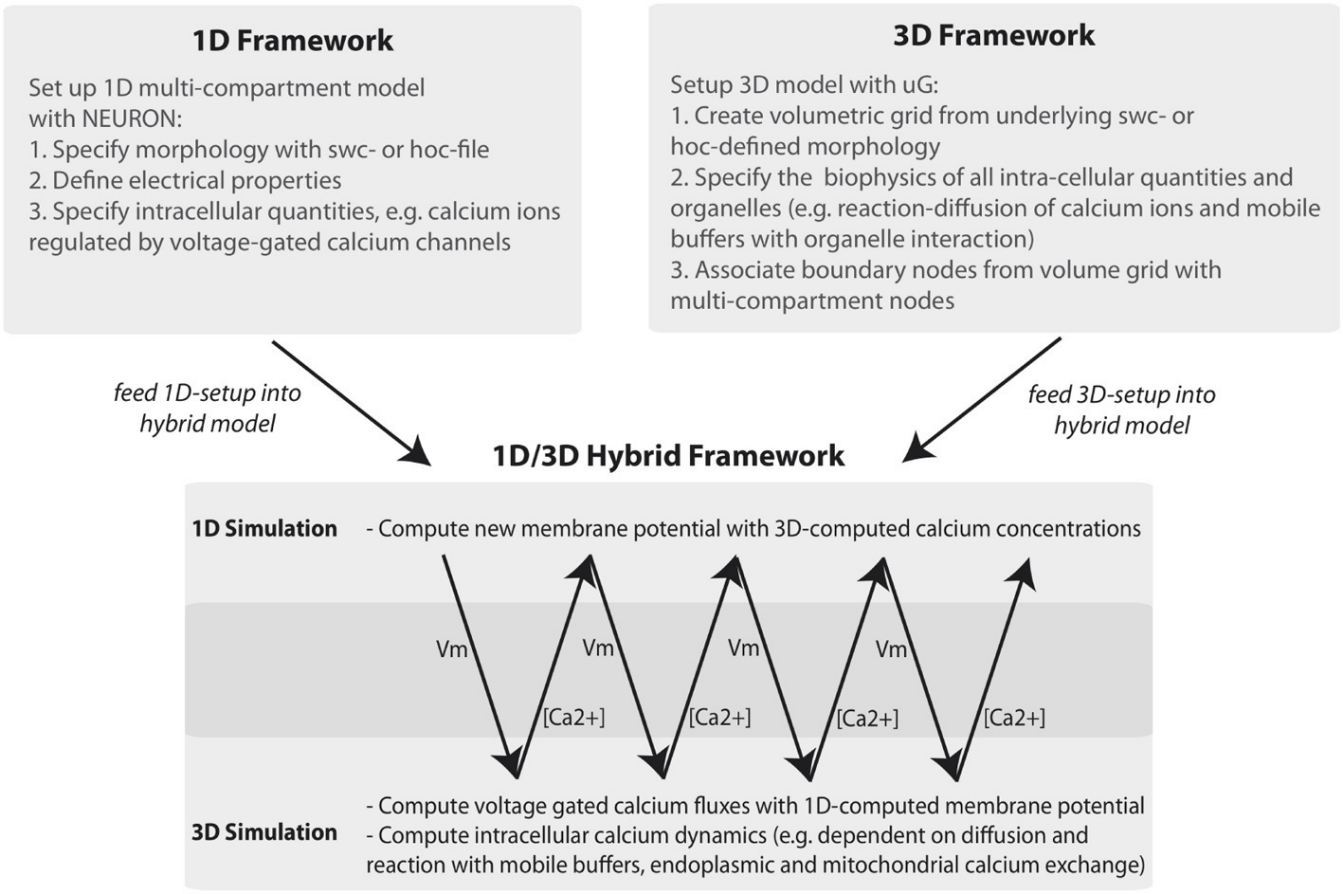
**Workflow of the 1D/3D hybrid framework**. The workflow can be separated into 1D and 3D modeling and simulation and into a set-up and simulation phase. In the set-up phase a general purpose simulator is used to define the one-dimensional problem (e.g., a hoc-geometry and set-up file). The 3D setup consists of generating a three-dimensional representation of the defined neuron and of specifying the cellular and intra-cellular components of the model problem. In the simulation phase 1D membrane potential simulation data is computed and mapped to the 3D framework. There, the membrane potential data is included in simulating intracellular processes, such as calcium signaling. The computed calcium data is then mapped back to the 1D problem, where it is used to compute calcium-dependent membrane fluxes.

The general workflow of the 1D/3D coupling is depicted in Figure 1. Using the graph-structure data contained in neuron compartment geometry files, we generate an equivalent 3D geometry with the compartment model graph as its “backbone.” This procedure is fully automated and quality controlled and will be discussed in a forth-coming paper. Since our presented framework is not limited to the grid generator used here, we would like to acknowledge the efforts of other authors addressing surface grid generation from point-diameter information, (e.g., McDougal et al., 2013b).

**Figure 2:**
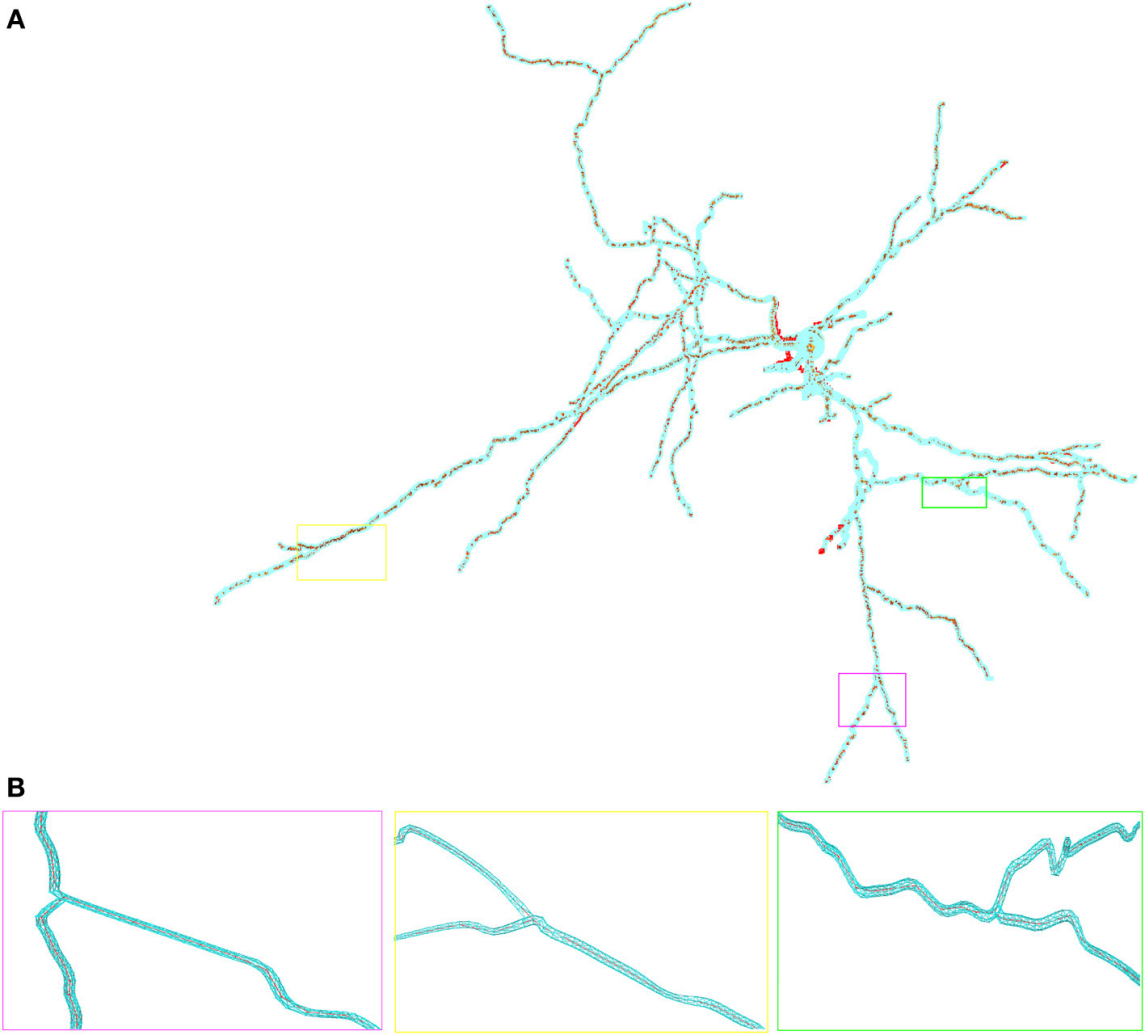
**Overlay of the multi-compartmental model defined by NEURON (red) and the equivalent tetrahedral volume mesh by uG (blue). (A)** View of the whole neuron from Katona et al. (2011). Note, that close to the soma local optimization of the morphology leads to slight divergence between NEURON and uG morphology. Optimization is performed when the multi-compartment geometry model would cause unphysiological intersections of dendrites at the soma in 3D, due to the fact that all dendrites in the multi-compartment description share a common soma node. **(B)** Magnified views of ROIs color-coded by rectangular boxes within the overview for comparison, showing the compartment model geometry defining the backbone of the generated surface grid.

In Figure 2 we show the in hoc-style defined neuron overlayed by the corresponding three-dimensional neuron, which can be used for the full-spatial simulation of intra-cellular processes. The quality of the volume grid influences the numerical accuracy and stability. In particular the tetrahedral angles are a means of determining the grid quality (Deuflhard and Weiser, 2011). For the grid used in this paper we measured minimal and maximal aspect ratios of the the triangular surface grid *AR_tri_* and the tetrahedral volume grid *AR_tet_* respectively, yielding values between 0.14 ≤ *AR_tri_* ≤ 0.86 and 0.21 ≤ *AR_tet_* ≤ 0.8. As a guideline one can state that aspect ratios above 0.1 result in grid elements that do not affect numerical stability (Shewchuk, 2002; Deuflhard and Weiser, 2011; Thompson et al., 2012).

### 2.3. Membrane potential mapping—*V_m_2uG*

In 1D compartment models the membrane potential *V_m_* is computed in one node per cylindrical compartment. In the 3D setting each original cylinder is now represented by a segment of the triangular surface grid, thus the membrane potential from one cylinder node needs to be mapped onto a calculated cluster of nodes in the triangular surface grid.

Associating each grid point $\vec{y}$ : = (*y*_1_, *y*_2_, *y*_3_) of the 3D morphology with compartment model grid points $\vec{x}$ : = (*x*_1_, *x*_2_, *x*_3_) (Figure 1) and the corresponding membrane potentials *V_m_* over the time-course of a simulation thus requires a mapping

(8) $$\begin{array}{l} V_{m}2uG:{\mathbb{R}}^{3}\times {\mathbb{R}}^{+}\times \mathbb{R}\to {\mathbb{R}}^{3}\times {\mathbb{R}}^{+}\times \mathbb{R}\\ \;(\vec{x},t,V_{m_{x}})\mapsto (\vec{y},t,V_{m_{y}}) \end{array}$$

that assigns a membrane potential *V_m_y__* to every grid point $\vec{y}$. *V_m_y__* is set to the potential *V_m_x__* associated with the nearest neighbor $\vec{x}$ : = (*x*_1_, *x*_2_, *x*_3_) of grid point $\vec{y}$ with respect to all points contained in the hoc-file in every timestep *t* of the simulation. The distance measure for calculating the nearest neighbor can be any Minkowski metric and is interchangeable. In this study we used Algorithms 1 and 2 and chose the euclidean metric, or *L*_2_-norm, such that

(9) $$\text{dist}(\text{p},\text{q}):={\left(\sum_{i\;=\;0}^{d\;-\;1}{\left(p_{i}-q_{i}\right)}^{2}\right)}^{1/2}$$

**Figure 3:**
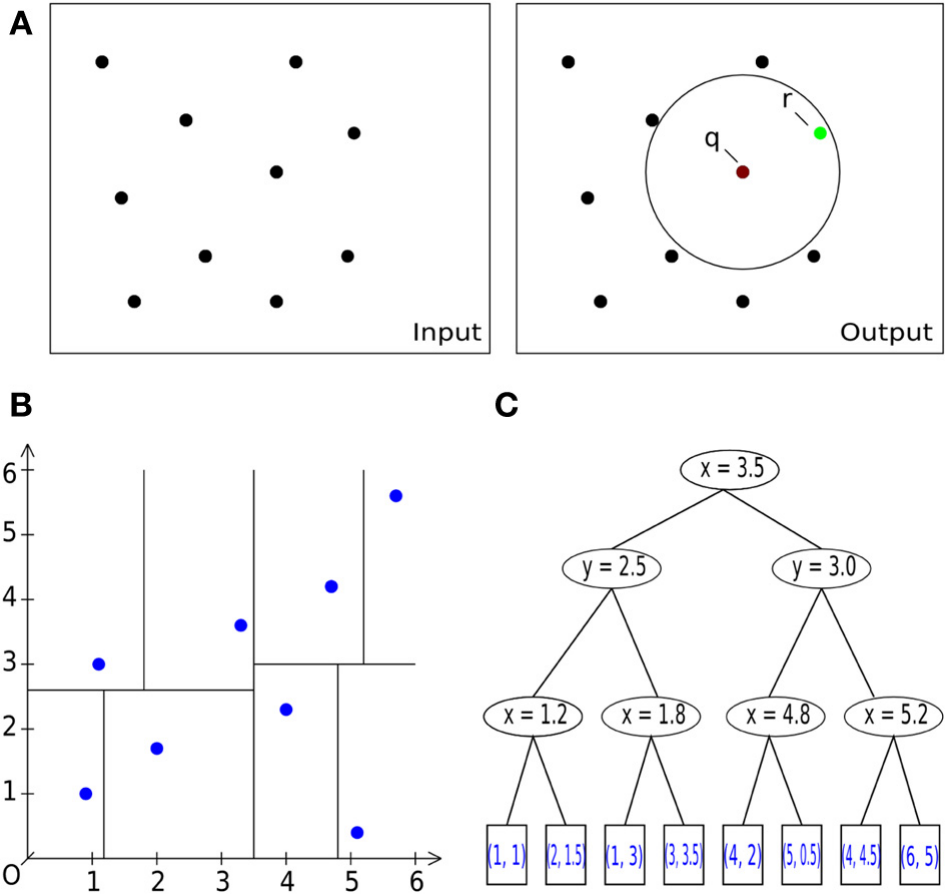
**Basic operations. (A)** General description of the nearest neighbor search task in 2D/3D: Let *S* be a set of #*n* points with dimension *d*: = 2, 3. Let *q* be a query point. The task is to find a point *r* (green) in the set *S* of all points (black), which is closest to the query point *q* [center of sphere in **(A)** on the right] within a given bounding box, i.e., within a circle (black) or sphere. **(A)** Pairwise comparison of all points in a radius search. B: Space-partitioning approach using k-d trees (multi-dimensional binary search trees). **(C)** An exemplary multi-dimensional binary search tree of dimension *d* = 2 (enhanced and modified according to Wikipedia, 2012), representing the space partition in **(B)**.

Because the geometry information provided by the hoc-file may be very large, i.e., the number of provided points *n* is large, we implemented and tested two strategies for determining the nearest neighbor. The first is an exact solution of the problem by space partitioning with multi-dimensional binary search trees, k-d trees with a search complexity 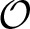
(*n* · log(*n*)) (see Bentley, 1975 as well as Figure 3 for an example), the second strategy is the exact solution by pairwise comparison with a search complexity 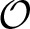
(*n*^2^). Note that we consider a three-dimensional example here, but the mapping process is dimension-independent and can be done for arbitrary dimensions by means of the underlying C++ library for multi-dimensional (binary) search trees (Mount and Arya, 2012), a library that is used in **V_m_*2uG*.

**Algorithm 1:** **Linear interpolation**.

q ← query point
2: NN ← getNearestNeighbor(q)
iDist ← NN.getDistance()
4: **if** *dist* ≤ *cutoff* **then**
NNs ← getNearestNeighbors(k)
6: **for** *i* = 1 → *k, j* = 1 → *k* ∧ *i* ≠ *j* **do**
g ← constructLine(NNs[i], NNs[j]) ▹ create straight line
8: q′ ← projectOnLine(q, g) ▹ orthogonally project onto line
**if** getDistance(q′, q) ≤ *iDist* **then**
10: iDist ← getDistance(q′, q)
Vm ← g.interpLinear(q′) ▹ linear interpolation
12: **end if**
**end for**
14: **end if**

**Algorithm 2:** **Bilinear interpolation**.

q ← query point
2: NN ← getNearestNeighbor(q)
iDist ← NN.getDistance()
4: **if** *dist* ≤ *cutoff* **then**
NNs ← getNearestNeighbors(k)
6: **for** *i* = 1 → *k, j* = 1 → *k*,
*l* = 1 → *k* ∧ *i* ≠ *j* ≠ *k* ≠ l **do**
det ← calculateDeterminant ▹ calculate determinant
8: p ← constructPlane(NN[i], NN[j],
NN[k], NN[l]) ▹ create plane
q′ ← projectOnPlane(q, p)
10: **if** getDistance(q′, q) ≤ *iDist* **then** ▹ orthogonal project onto plane
iDist ← getDistance(q′, q)
12: Vm ← p.interpBilinear(q′) ▹ bilinear interpolation
**end if**
14: **end for**
**end if**

### 2.4. Algorithms

The pairwise comparison is an exhaustive search that requires no additional data structure but 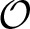 (*n* · *m*) iterations which is impractical if the number *n* of provided points on the grid is large, and for the number of corresponding points in the hoc file holds *n* ≈ *m*, thus arriving at quadratic runtime complexity 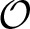
(*n*^2^). For large *n*, we therefore use the method of space partitioning (k-d trees, Figures 3B,C) which leads to an improved average runtime complexity for a query of $\text{log}\left(\frac{n}{d}\right)$, with *d* being the space dimension. With this approach we reach super-linear runtimes after the initial tree has been built. In contrast to the 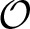
(*n*^2^) method, we need an additional structure, which is justified because of the overall speedup compared to the naïve approach. The overall runtime is dominated by the utilized search algorithm used during the initial build of the tree and the balancing of the tree itself, yielding a complexity 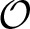
(*n* · log(*n*)) (Wald and Hvran, 2006; Cormen et al., 2011). The worst case arises if *n* « 2^*d*^ is satisfied and the runtime complexity increases per query to 
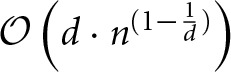
 (Lee, 1997). Yet, the curse of dimensionality is not an issue in the presented application, since our geometry is defined in three-dimensional space.

*V_m_2uG* offers linear or bilinear membrane potential interpolation (Algorithms 1 and 2). This can be useful if the compartmental representation of the neuron is very coarse and mapping distances become large, i.e., 3D surface grid points lie far away from the computed nearest neighbor on the compartment geometry. Interpolation is then carried out over *n* ∈ ℕ^+^ pseudo nearest neighbors to compute an optimal approximated value for the membrane potential which is then assigned to the given grid point.

### 2.5. Workflow and bidirectional coupling

**Figure 4:**
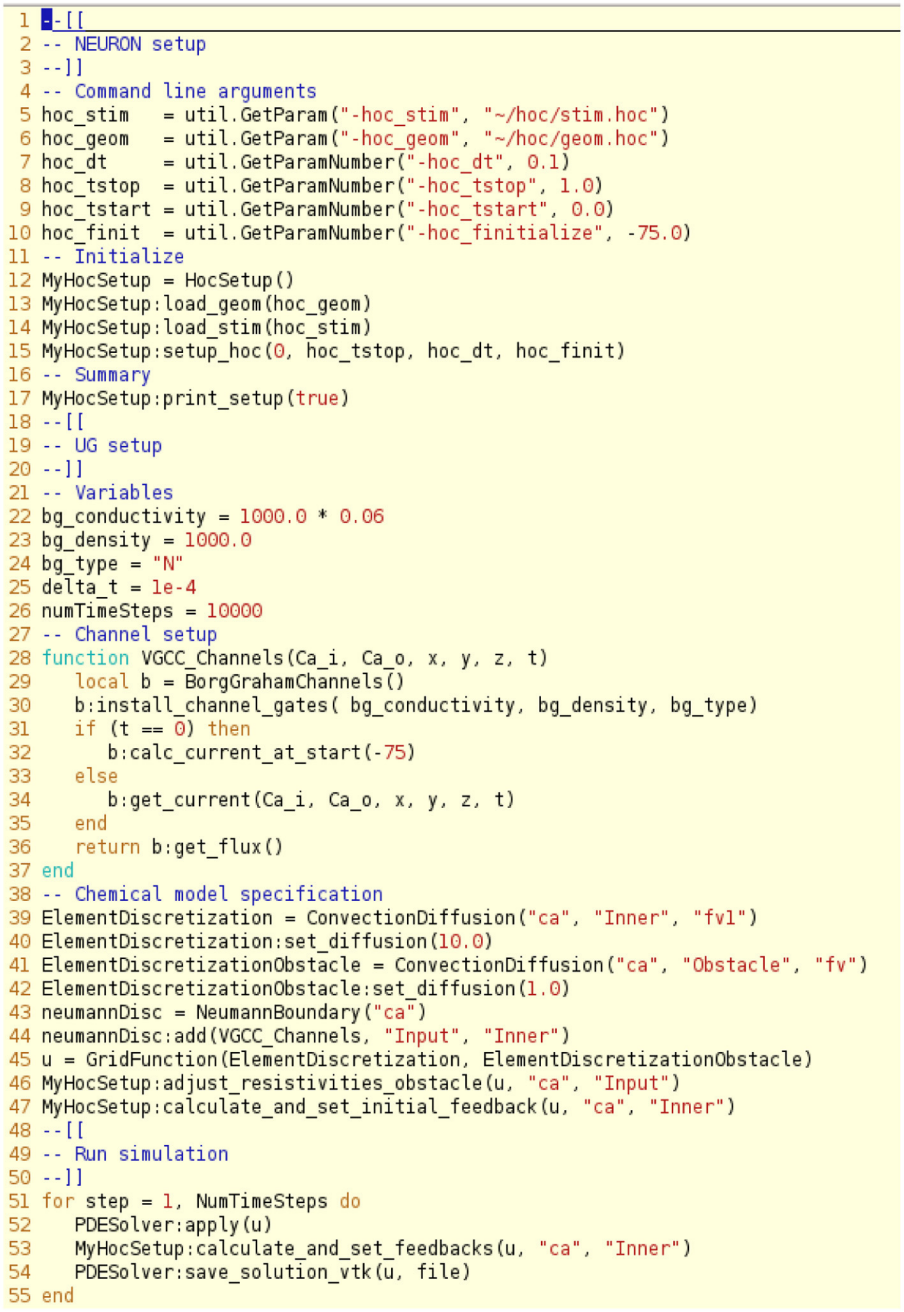
**Script file illustrating the setup of the NEURON and uG model, here divided into a NEURON setup phase, a uG setup phase, chemical model specifications and the simulation run control**. One can make use of the hoc interpreter features within the lua script files that define the uG-setup. Note that one can specify the geometry and stimulation protocol in distinct files and combine this with using the hoc interpreter from uG to refine the setup by statements of the form: *HocSetup:execute(“command string”)*.

We use a direct data coupling mechanism termed a *type A problem* in Heterogenous Multiscale Modeling, see (Weinan et al., 2003), utilizing an online algorithm within uG. The NEURON library is used as a plugin/shared library within the uG project. The simulation workflow is defined in uG by a lua-script (Ierusalimschy et al., 2013) (see Figure 4 for the script used in this paper). We provide fully flexible control over NEURON within uG by means of a custom API or by directly including the hoc-script (which is then executed by the HOC language interpreter). For the latter we developed an additional C++ wrapper.

The workflow defined in the lua-script performs the steps illustrated in Figures 1, 4. The numerical procedures are defined individually for NEURON and uG (see Materials and Methods), which is necessary since the model equations for the 1D and 3D model are of different types. The only coupling requirement is that both simulations can synchronize their data at specific time points, which is guaranteed by our framework.

Data exchange between 1D and 3D model is bidirectional, where membrane potential data is passed from 1D to 3D via the mapping algorithm presented in the previous section. The computed ion concentrations in the 3D model are then passed from 3D to 1D in the following way:

Consider a cylindrical compartment *C_i_* of the 1D model. By ∂(*C_i_* ∩ ∂Ω) we denote the portion of the cell surface ∂Ω that is associated with compartment *C_i_*. We can then compute the intra-cellular calcium concentration for *C_i_* as

(10) $$\begin{array}{l} \left[Ca^{2+}]C_{i}=\frac{\left\|\partial C_{i}\right\|}{\left\|\partial (C_{i}\cap \partial \Omega )\right\|}\int_{\partial (C_{i}\cap \partial \Omega )}u_{Ca^{2+}}(\vec{x}\right)\\ \text{ for }\vec{x}\in \partial (C_{i}\cap \partial \Omega )\text{ and }\forall i=0,\ldots ,N \end{array}$$

where *u*_*Ca*^2+^_($\vec{x}$) is the calcium concentration in node $\vec{x}$ of the 3D grid and *N* is the number of compartments representing the 1D geometry. ∥.∥ symbolizes the size of ∂ *C_i_* and ∂(*C_i_* ∩ ∂Ω) respectively.

The factor $\frac{\left\|\partial C_{i}\right\|}{\left\|\partial (C_{i}\cap \partial \Omega )\right\|}$ accounts for the fact, that the surface size of ∂(*C_i_* ∩ ∂Ω) and the surface of *C_i_* are not necessarily identical. Computing the calcium fluxes in uG and in NEURON using the values computed by Equation (10) shows good agreement between the two models, see Supplemental Figure S2 as well as Supplemental Figure S3.

### 2.6. Simulations using the 1D/3D hybrid method

**Figure 5:**
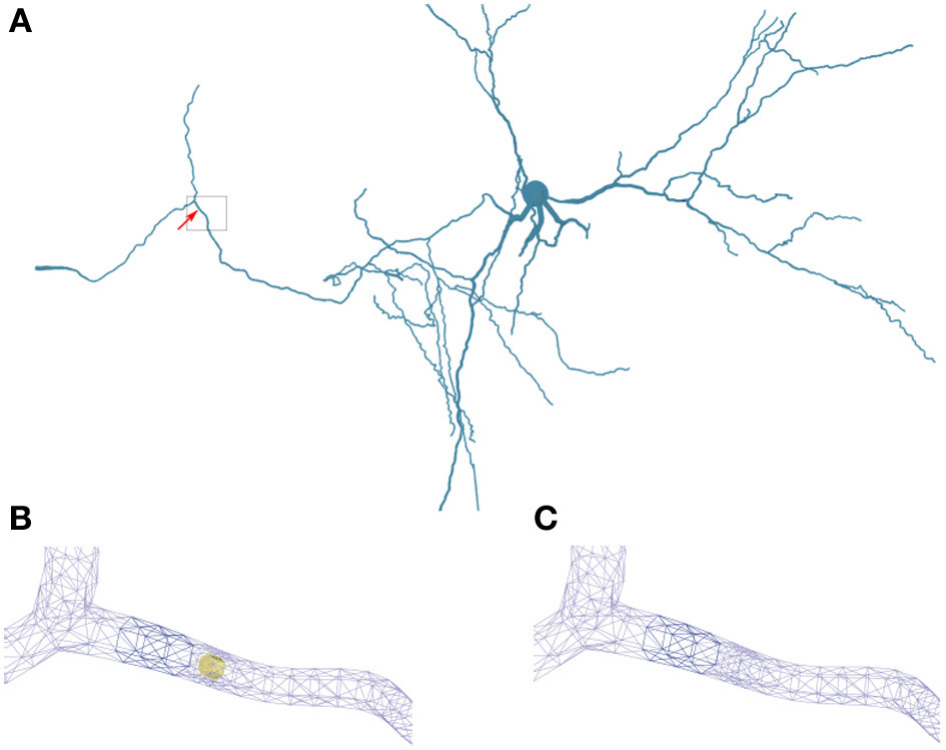
**Geometry specifications. (A)** Three-dimensional neuron from Katona et al. (2011) used in the simulations. The box highlights the region of interest used in **(B,C)**. **(B)** Triangular surface grid of a dendritic segment including an intracellular obstacle (yellow). The arrow in **(A)** indicates the position of the obstacle. **(C)** Triangular surface grid of a dendritic segment without the intracellular obstacle. The surface grid can remain unaffected by the insertion of an intra-cellular object.

We applied the described method for coupling 1D and 3D simulations to a CA1 interneuron from Katona et al. (2011), taken from the database ModelDB (Migliore et al., 2003). We set up a NEURON simulation with stimulation protocol (see Materials and Methods) and generated a three-dimensional geometry for calcium simulations that use the membrane potential data from the NEURON simulation (Figure 5) and feeds back the computed concentrations (see previous section). An overlay of hoc- and ugx- (the uG grid format) geometry shows good agreement between the two.

The scientific gain from 3D simulations lies in a physically detailed representation of morphology and the underlying biophysical processes in neurons. These processes are regulated by space and time-dependent variations of parameters like channel densities, cytosolic and organelle architecture, diffusivity and of course cellular and organelle morphology. Previous work has demonstrated the importance of investigating the spatial organization of ion channels and their stochastic behavior, (Oliveira et al., 2010; Brandi et al., 2011), in part using a multi-scale modeling approach. Brandi et al. (2011) for instance present in their abstract a platform for combining NeuroRD (Blackwell et al., 2013) and MOOSE (Ray and Bhalla, 2008). The framework employed here can be viewed as a natural extension of previously published multi-scale approaches, with a strong focus on resolving the intra-cellular space with additional accuracy, rather than interpreting the intra-cellular space as a homogenous space for reaction-diffusion processes. Obstacles or active organelles, such as calcium-relevant stores (e.g., the endoplasmic reticulum or mitochondria), can be easily integrated into our hybrid framework. In addition to this, our framework aims at simulating entire neurons up to small networks with a high level of cellular and intra-cellular detail, rather than investigating e.g., the single molecule level by Smoldyn Andrews et al. (2010). To accomplish this, we model at the continuum scale and make use of the massively scalable code uG, (Heppner et al., 2013), demonstrated to perform ideal weak scaling up to 64 k processes on the Hermit Supercomputer. In Table 2 we show ideal weak scaling for the presented simulations on our in-house cluster (see Materials and Methods), solving a 3D problem with 256,000 grid points in under 1 min on 64 processors. In comparison to other simulators, such as VCell or MCell, the VCell website lists a 2D benchmark with a problem size of 5000–10,000 grid points and a PDE-system with 4–5 PDEs being solved in roughly 20 min. In Balls and Baden (2004), MCell scaling studies up to 64 cores show a scaling efficiency of 85–92%, solving a benchmark problem also in roughly 20 min. Since the underlying models of the compared simulators differ, a direct comparison is not necessarily valid. The presented hybrid method, based on a continuum model is applicable ideally for whole- and multi-cell simulations, while e.g., MCell uses particle-based methods that so far do not go beyond spine simulations, i.e., microdomain simulations.

**Table 2:** **Scaling**.

**Problem size [# nodes]**	**Procs [#]**	**Runtime [s]**
4000	1	59
16,000	4	58
64,000	16	47
128,000	32	49
256,000	64	49

*The 1D/3D hybrid framework exhibits weak scaling on the in-house cluster (32 nodes with each providing an Intel Core i7 CPU @ 2.67 GHz running Linux). We solved the presented model equations using a Newton solver for the non-linear part and used a BiCGStab method as iterative solver combined with ILU-smoothened geometric multigrid preconditioner. Each runtime is the average of *n* = 10 runs each, where each run was a simulation of 1/10 s real time and a numerical time step width of 1 ms*.

In order to demonstrate some advantages of a 1D/3D hybrid approach, we defined a standard 1D/3D simulation protocol (see Materials and Methods) and then varied parameters, such as calcium diffusivity (Allbritton et al., 1992), voltage-gated calcium channel (VGCC) densities (Pumplin et al., 1981; Eggermann et al., 2011), and added intra-cellular obstacles to the cytosol.

**Figure 6:**
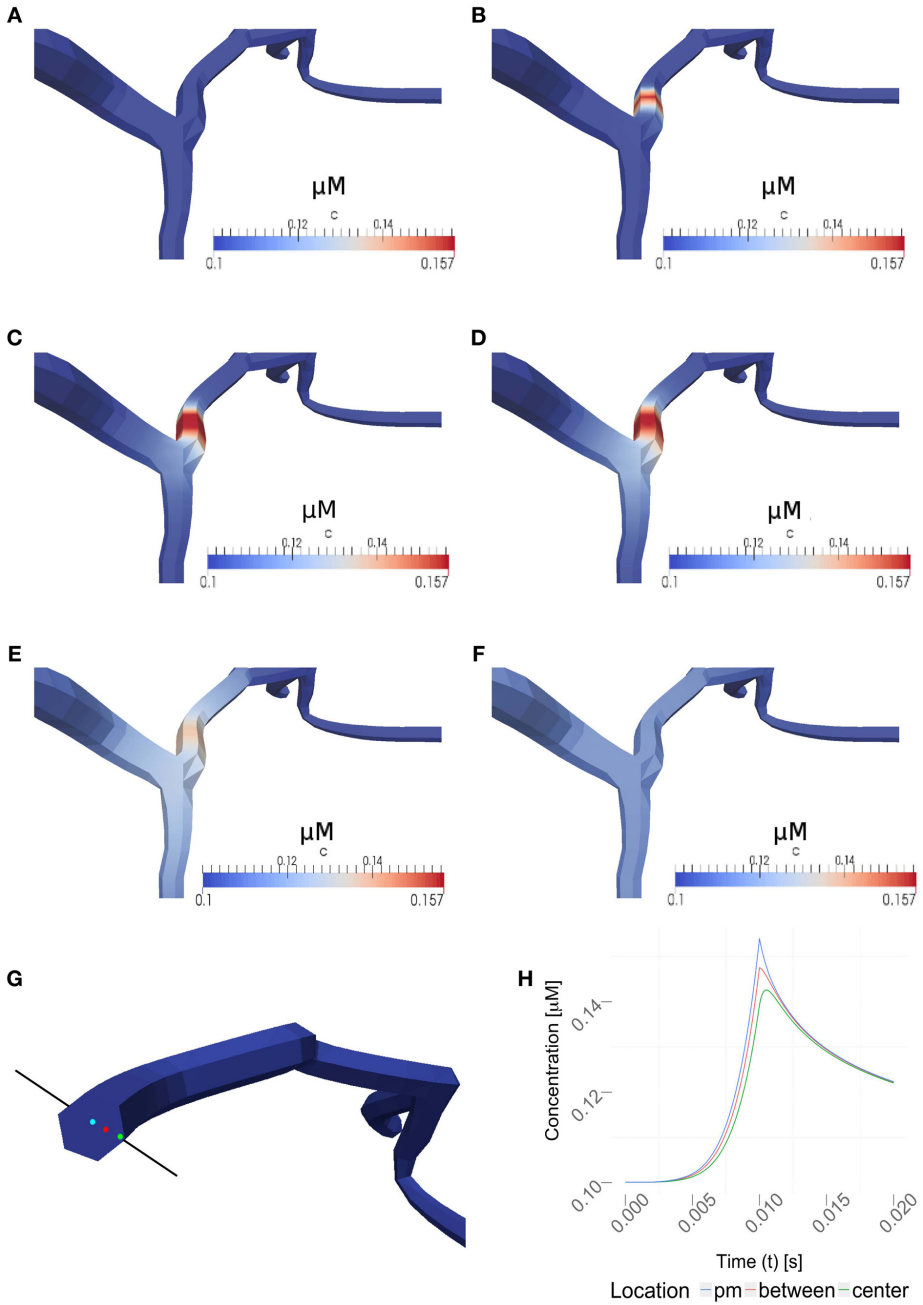
**Time series for a simulation of intra-cellular calcium signaling with a calcium diffusion coefficient set to 100 μm^2^/*s*, VGCC density of 1000 μm^−2^ and no obstacle present in the dendrite**. The basal calcium concentration is set to 100 nM. **(A–F)** show the propagation of calcium in a dendrite, triggered by the the stimulation protocol listed in Table 1. Membrane potentials were computed in NEURON and mapped to the plasma membrane in order to compute the VGCC-dynamics regulating the calcium exchange. Calcium concentrations were then mapped back to compute the calcium-dependent membrane potential in NEURON. **(A–F)** covers 1s of real time. Time points shown are at 0, 0.01, 0.02, 0.03, 0.1, and 1 s. Note, the maximum single-channel conductance was set to 60 pS according to Graham (1999). **(G)** Highlighted measuring points close to the plasma membrane (pm) in between and at the center used in **(H). (H)** Evolvement of the calcium concentrations at three different points over a period of 20 ms. Note that the VGCCs close at the peak amplitude and the calcium profile quickly adjust to a uniform value based on the intra-cellular diffusion.

Figure 6 and the supplemental movies show calcium simulations in a region of interest using the 1D/3D hybrid method, with the calcium diffusion set to 100 μm^2^/s, a VGCC-density of 1000 μm^−2^ and no obstacle present in the cytosol (see Figure 5 for the neuron geometry used in the simulations). This example shows how the NEURON-calculated membrane potentials are mapped to the 3D model, where they regulate VGCCs and detailed calcium dynamics can be simulated in full three-dimensional space. The calcium concentrations are then mapped back to NEURON according to the previous section.

#### 2.6.1. Varying the diffusion coefficient

**Figure 7:**
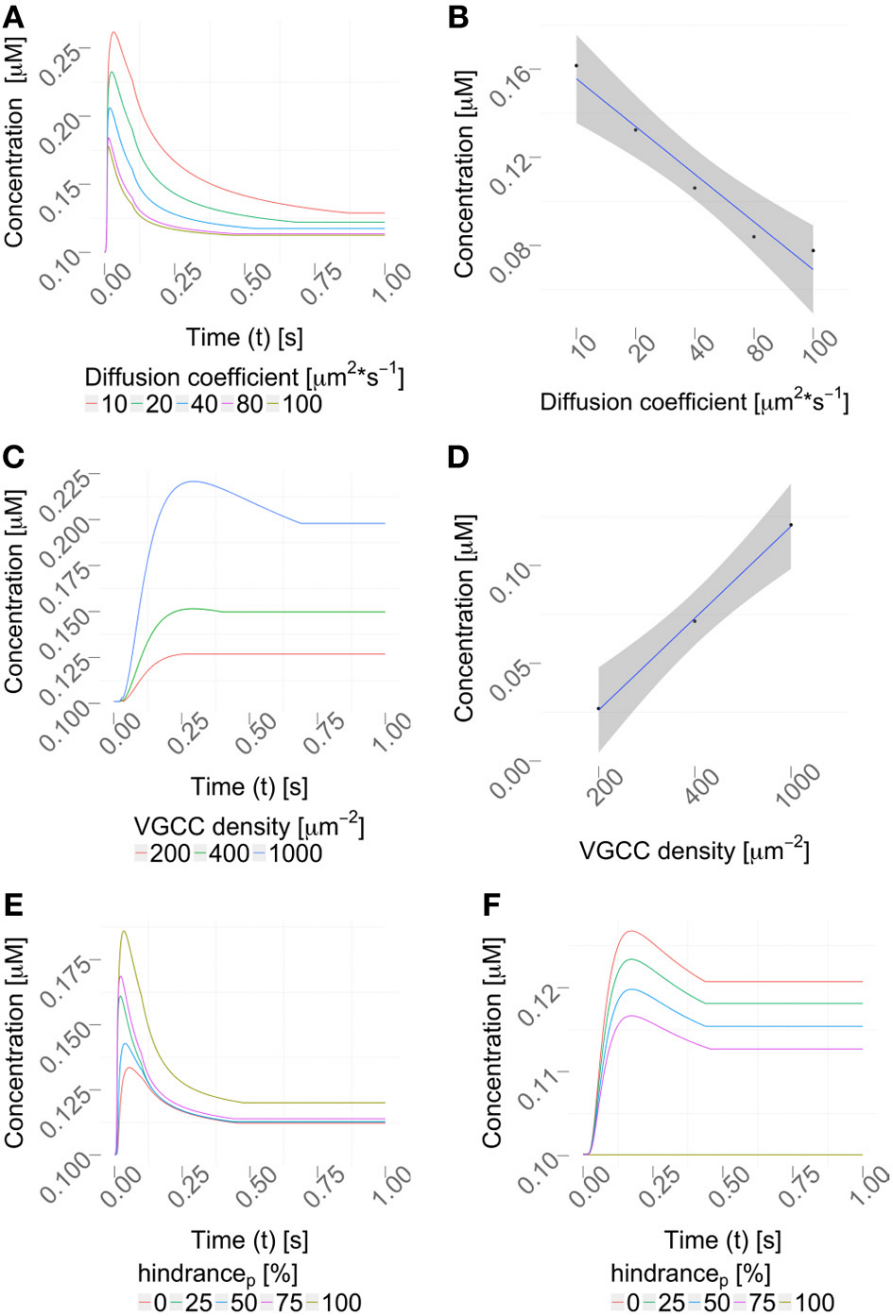
**Parameter variation. (A)** Variation of the diffusion coefficient regulates the intracellular calcium profile (using a constant VGCC density). The concentration profiles computed by the hybrid framework are validated in Figure S3 using an alternative, reductionist approach. **(B)** Quantification of the peak amplitudes in **(A)**, *R*^2^ = 0.9461 and *p* = 0.003, showing a linear dependency between the diffusion coefficient and the peak amplitude. **(C)** Density variation of VGCCs sharpens the concentration profile in the considered dendrite (using a constant diffusion coefficient). **(D)** Quantification of the peak amplitudes in **(C)**, *R*^2^ = 0.99 and *p* = 0.018 showing a linear dependency between VGCC density and peak amplitudes. Calcium profiles are validated by calcium imaging data, Helmchen et al. (1996); Kits et al. (1997); Maravall et al. (2000). VGCC densities are varied according to documented physiological intervals up to 10000/μm^2^, as reported in Eggermann et al. (2011). **(E,F)** Placing obstacles of various sizes in the dendrite, from 0 to 100 % (see Table 3) affects the locally available calcium concentration, **(E)** before the obstacle and **(F)** behind the obstacle.

We then varied the diffusion coefficient for intracellular calcium between 10 and 100 μm^2^/s, which is within the experimentally observed ranges, (Allbritton et al., 1992), while the VGCC density was set to 1000 μm^−2^ (Figure 7A). Figure 7B shows a linear dependence to a global variation of the cytosolic calcium diffusion coefficient. Note, that the diffusion of calcium does not have to be isotropic, but can have different diffusion properties in the three space-dimensions. The diffusion coefficient then needs to be formulated as a diffusion tensor

$$D:=\left(\begin{array}{ccc} d_{x} & 0 & 0\\ 0 & d_{y} & 0\\ 0 & 0 & d_{z} \end{array}\right),$$

which can be defined in the uG-workflow (Vogel et al., in press).

#### 2.6.2. Varying channel densities

Next, we decided to vary the VGCC densities on the plasma membrane. We placed N-type VGCCs (see Materials and Methods) homogeneously along the dendrites at densities between 200 and 1000 μ*m*^−2^ (Figure 7C). Due to an increased channel density we observe changes in the rise and decay times, as well as different peak amplitudes in the calcium profile. In Figure 7D we see that the peak amplitude of the intra-cellular calcium signal depends linearly on the VGCC density. Global and time-dependent changes in the VGCC distribution of the neuron can thus finely regulate the intra-cellular calcium code.

#### 2.6.3. Including intra-cellular obstacles

Where in a 1D model one needs to average the intra-cellular space and approximate morphologies, the 3D approach allows a full and detailed investigation. To demonstrate how intra-cellular objects can be included into a simulation, we investigated calcium dynamics in the observed dendrite with and without an intra-cellular obstacle. For this we placed an obstacle (this could be e.g., a mitochondrion or endoplasmic reticulum) in the center of a dendrite (see Figure 5), defined as a cylinder that is 0.14 μm long and has a variable circumference *C_obstacle_*, embedded in a dendrite of average local circumference *C_neurite_*. We then define the *hindrance factor H* of the intracellular *obstacle* with respect to the surrounding *cylinder* for that particular section as:

(11) $$H:=\frac{C_{obstacle}}{C_{cylinder}}\in \;[0,1]$$

The obstacle sizes used in our simulations are listed in Table 3, which correspond to a dendritic occupancy between 0 and 100% (100% corresponds to a full blockage in the the dendrite). We chose the obstacle location to be right before the most distal bifurcation point of a certain neurite which emerges—and can be visually traced in Figure 5A—from the left most bottom of the soma itself. Note, that the obstacle is placed in the middle of the considered neurite in the present use case. Intra-cellular organelles, occupying parts of the cytosol, affect the intra-cellular resistivity. To account for this in our 1D model, one can either directly modify the axial resistance by changing the intra-cellular resistance or by changing the diameter of the cylinders that harbor an organelle. The axial resistance *R_a_* in a cylinder with length *l* and diameter *d* is computed by:

(12) $$R_{a}=R_{i}\cdot \frac{4l}{d^{2}\pi }$$

where *R_i_* is the intra-cellular resistance. The effective diameter can be calculated for all affected compartments by:

(13) $$d_{new}=d_{old}-d_{obstacle}$$

**Table 3:** **Hindrance factors**.

**Hindrance [%]**	**Obstacle volume [μm^3^]**	**Obstacle surface area [μm^2^]**
0	0.0000	0.0000
25	0.0022	0.3040
50	0.0344	1.9000
75	0.0945	3.7240
100	0.4076	8.6401

*Listed above are the surface and volume sizes of the obstacles that were placed in the dendrite for the simulations shown in Figure 7 with corresponding hindrance factors. A hindrance factor of 100 % represents a full blockage of the dendritic cross-section*.

Our framework automatically checks which compartments are occupied by organelles and adjusts the diameters accordingly. We observed that local intracellular signaling is affected by obstacle size and position (Figures 7E,F). In more complex situations, e.g., space occupancy by mitochondria, endoplasmic reticulum or spine apparatuses in dendrites or spine necks, the inclusion of these functional organelles in a three-dimensional model might be necessary for explaining the intracellular dynamics of the neuron. Active intra-cellular organelles can be easily added within the presented framework. The user needs to define the geometry of the organelle (which can be a detailed three-dimensional reconstruction) and the biological exchange mechanisms across the organelle membrane (e.g., IP3-receptors, ryanodine receptors, SERCA pumps, sodium-calcium exchangers etc.).

## 3. Discussion

Modeling and simulating cellular and network processes are constrained by the complexity of the computational problem and the availability of experimental data to validate the models. Experimental techniques are evolving, shedding new light on the filigreed functioning of neurons and networks. Spatial and time resolutions are becoming fine enough to resolve positions of single proteins and receptors at synapses and the intra-cellular domain can be investigated at increasing levels of detail. On the other hand computational complexity increases when a model includes biological and spatial detail, resulting in a highly detailed three-dimensional model of neurons. Computational complexity further increases when not only one neuron, but entire networks need to be modeled.

To cope with network complexity, several approximation methods for cellular and network function have been developed over the last decades. These approximations yield zero- or one-dimensional models in space, describing neurons with point- or compartment neuron models. Numerous specialized and general purpose neuron simulators incorporate these basic abstraction techniques (e.g., Bower and Beeman, 1997; Hines and Carnevale, 2003; Gewaltig and Diesmann, 2007) and allow the user to simulate large and complex neural network structures. These models and simulators were developed and evolved not only around the limitations of computational power, but also around the availability of experimental data to validate the models. The evolution of experimental methods, computational resources and numerical mathematics motivates the development of neuron models that include greater physical detail. This brings us to three-dimensional models that include the detailed morphology of neurons, either by modeling physical processes using stochastic (e.g., Andrews et al., 2010; Oliveira et al., 2010) or deterministic models. We make use of the latter approach with the additional feature of being able to integrate obstacles and intracellular organelles, which yields models formulated on the continuum scale by means of systems of partial differential equations. Solving these equations numerically requires advanced mathematical tools. One general purpose platform is uG (Bastian et al. 1997), which we used in this paper. uG offers numerical discretization methods and efficient solvers for systems of partial differential equations on highly unstructured grids and runs on massively parallel systems (Heppner et al., 2013; Vogel et al., in press). As shown in the past, these numerical tools are applicable and efficient on a cellular level or in a networks with relatively small numbers of neurons (Xylouris et al., 2007; Wittmann et al., 2009; Xylouris, 2013).

Given the limitations in computing resources, it is currently not feasible to model and simulate large networks of neurons with full single cell, three-dimensional detail. To make use of the advantages of highly detailed three-dimensional models and to cope with the complexity that comes with modeling large networks of neurons, we developed a method to couple state of the art general purpose neuron simulators, e.g., NEURON, with three-dimensional models of single neurons. This 1D/3D hybrid method includes automated tools to either reconstruct three-dimensional neuron morphologies from raw microscopy data (Broser et al., 2004; Queisser et al., 2008; Jungblut et al., 2011), from anatomically recorded data (Wolf et al., 2013) or from graph-structure morphologies as used, e.g., in the NEURON Simulator in the form of hoc-files or swc-files.

In this paper we introduced a framework for geometry and membrane potential and intra-cellular ion concentration mapping between 1D simulations and the equivalent 3D model. For this we used the NEURON simulator to compute electrical signals in a compartment neuron and uG to simulate intracellular calcium dynamics in 3D. With this approach we were able to exploit the modeling and computational advantages that a general purpose simulator for large networks brings, as well as the necessary tools to investigate intra-cellular (or even extra-cellular) processes on a very fine scale. By including the detailed morphology of neurons—which can be subject to temporal adaption due to neuronal activity (Silver et al., 1990; Korkotian and Segal, 1999; Muller et al., 2002; Van Aelst and Cline, 2004; Hayashi and Majewska, 2005; Tada and Sheng, 2006; Wittmann et al., 2009; Kanamori, 2013)—the interplay between cellular/organelle morphology and cellular function can be systematically investigated. Using defined stimulation protocols in NEURON, we ran 1D-simulations mapping the results onto the three-dimensional morphology as boundary conditions for a cellular calcium model and vice versa. The calcium model consisted of a local distribution of N-type voltage-gated calcium channels, regulated by the membrane potential and intracellular diffusion and reaction of calcium ions with a mobile buffer. After presenting the functionality of the 1D/3D hybrid method on a reference parameter set, we varied the density of voltage-gated calcium channels, the calcium diffusion coefficient and introduced intracellular obstacles. The results show that for one, the presented method is designed in a general fashion and is thus applicable to a broad range of neurobiological questions and that the effect of intra-cellular obstacles, locally changing channel densities and cytosolic diffusivity have a substantial effect on intracellular signals.

For future research, problem-specific models can be used within the presented framework. For instance, not only single neurons, but entire networks could be simulated on the 1D level, where a small subset of “key neurons” in the network can be resolved in full three-dimensional detail (Xylouris et al., 2007). Furthermore intra-cellular models for different ionic species, detailed models of intra-cellular organelles, such as mitochondria and endoplasmic reticulum calcium exchangers, protein synthesis and synapses could be included on the 3D scale.

## 4. Materials and methods

### 4.1. The 1D electrical model

To simulate the electrical activity of the model neuron, we used the classical Hodgkin-Huxley equations (Hodgkin and Huxley, 1952) including sodium, potassium, calcium and a leakage current:

(14) $$\begin{array}{l} i_{m}=c_{m}\frac{\partial V}{\partial t}+n^{4}{\tilde{g}}_{K}(V-E_{K})+m^{3}h{\tilde{g}}_{Na}(V-E_{Na})\\ \;+{\tilde{g}}_{L}(V-E_{L})+I_{Ca}, \end{array}$$

where *V_m_* is the membrane potential, *c_m_* the membrane capacity, $\tilde{g}$_*Na*_, $\tilde{g}$_*K*_ and $\tilde{g}$_*L*_ are the maximum single channel conductances for sodium and potassium channels, as well as a leakage current. *E_K_*, *E_Na_* and *E_L_* are the reversal potentials. *I_Ca_* is the calcium current specified by Equation (26). The time and voltage-dependent gating variables *n, m, h* determine the gating behavior of the channels and are computed by the ordinary differential equation

(15) $$\frac{\partial x}{\partial t}=\frac{x_{\infty }-x}{\tau_{x}}$$

where *x* = *n, m, h*. The parameters *x*_∞_ and τ_*x*_ in the equations above are computed by

(16) $$x_{\infty }=\frac{\alpha_{x}}{\left(\alpha_{x}+\beta_{x}\right)}$$

and

(17) $$\tau_{x}=\frac{1}{\alpha_{x}+\beta_{x}}+\tau_{x,0}$$

where τ_*x*, 0_ = 0 for *x* = *n, m, h*. The values for α_*x*_ and β_*x*_ are derived from (Hodgkin and Huxley, 1952):

(18) $$\alpha_{n}=\frac{0.01(V+10)}{e^{\frac{V-10}{10}}-1}$$

(19) $$\beta_{n}=\frac{e^{\frac{V}{80}}}{8}$$

(20) $$\alpha_{m}=\frac{0.1(V+25)}{e^{\frac{V+25}{10}}-1}$$

(21) $$\beta_{m}=4e^{\frac{V}{18}}$$

(22) $$\alpha_{h}=0.07e^{\frac{V}{20}}$$

(23) $$\beta_{h}=\frac{1}{e^{\frac{V+30}{10}}+1}$$

Solving a multi-compartment model where for each cylindrical compartment an equation of the type

(24) $$\frac{dV_{m}}{dt}=-i_{m}+I_{electrode}$$

is computed using the NEURON simulator. Numerically, the arising sets of ordinary differential equations are solved with the Crank-Nicolson method implemented in NEURON. We chose the CA1 stratum radiatum hippocampal interneuron published in Katona et al. (2011) and ran the following stimulation protocol:

We inserted a current clamp at the mid of the soma and stimulated with amplitude of 0.1 nA with timestep *dt* = 0.1 ms for a total time of 10 ms. We then simulated 1 s of membrane potential propagation in NEURON evoked due to that stimulation.

### 4.2. Voltage-gated calcium channel models

In order to include voltage-gated calcium channels, we used the Borg-Graham model described for different ion channel types in detail in Graham (1999). In our setup we included N-type calcium channels gates, though the implementation allows us to also include other types (e.g., L-/T-type gates). We define a mapping of the calcium fluxes calculated by the VGCC-model onto the 3D morphology:

(25) $$\text{Borg Graham}:{\mathbb{R}}^{3}\times {\mathbb{R}}^{+}\times \mathbb{R}\to \mathbb{R}:(x_{b},t,v_{m_{b}})\mapsto F$$

The dynamics of calcium ionic fluxes are described in the following way (Graham, 1999) and are computed inside the 1D-simulation loop:

(26) $$I_{Ca}(V,t,{\left[Ca\right]}_{i},{\left[Ca\right]}_{o})=G(V,t)F(V,{\left[Ca\right]}_{i},{\left[Ca\right]}_{o})$$

*G* describes the properties of the gating functions, in particular the open and close state-probabilities of the channels and is specified in Equation (28). *F* can be computed using the GHK model, yielding

(27) $$\begin{array}{l} F(V,[Ca]i,[Ca]o)\\ \;=p_{Ca}\frac{z^{2}F^{2}V}{RT}\left[\frac{{\left[Ca^{2+}\right]}_{i}-{\left[Ca^{2+}\right]}_{o}\exp\text{​}(-z\frac{FV}{RT})}{1-\exp\text{​}(-z\frac{FV}{RT})}\right] \end{array}$$

where *p_Ca_* is the permeability and for N-type calcium channels is set to the value 10^−8^ *cm*^3^/*s*. *F* denotes the Faraday constant, *R* the gas constant and *T* the temperature. For the VGCCs used in the simulations, the gating function *G* is defined as

(28) $$G(V,t)=k(V,t)l^{2}(V,t)$$

Here, *k* and *l* fulfill the ordinary differential equation (15) and eqs. (16, 17), using the following values:

(29) $$\begin{array}{l} \alpha_{x}(V)=K_{x}\exp\left(\frac{z_{x}\gamma_{x}(V-V_{1/2,x})F}{RT}\right)\\ \beta_{x}(V)=K_{x}\exp\left(\frac{-z_{x}(1-\gamma_{x})(V-V_{1/2,x})F}{RT}\right) \end{array}$$

The parameters *V*_1/2_, *z*, γ, *K*, τ_0_ also depend on the particular channel and are documented in Graham (1999). We set the values accordingly:

$$\begin{array}{l} V_{1/2,k}:=-21\text{m}\\ \text{ V}z_{k}:=2\\ \;\gamma_{k}:=0\\ \;K_{k}:=1.7\text{ms}\\ \;\tau_{k,0}:=1.7\text{ms}\\ V_{1/2,l}:=-40\text{m}\\ \text{ V}z_{l}:=1\\ \;\gamma_{l}:=0\\ \;K_{l}:=70\text{ms}\\ \;\tau_{l,0}:=70\text{ms} \end{array}$$

It is sufficient to solve Equation (15) using the first order explicit Euler method, in which the required membrane potential is taken from the membrane potential mapping (*V_m_2uG*) in each time step.

### 4.3. Numerical approach

The calcium model used in the presented simulations is based on a cytosolic diffusion equation with boundary conditions that include the plasma membrane calcium exchange mechanisms, i.e., Neumann boundary conditions representing an ionic flux over a given surface area of the plasma membrane. This can be stated as:

(30) $$\nabla \cdot (D\nabla u(\vec{x},t))=\frac{\partial }{\partial t}u(\vec{x},t)\text{ in }\Omega \subset {\mathbb{R}}^{3}$$

with boundary conditions

(31) $$\frac{\partial u}{\partial n}=g(Channels)$$

where *u* denotes the intracellular calcium concentration, *D* the diffusion tensor (a 3 × 3-Matrix) and *g(Channels)* the calcium flux depending on the channels defined in the simulation (see previous section). To solve Equation 30, we discretized in space by means of first order finite volumes, treating the time derivative by an implicit Euler method.

For this we discretized the plasma membrane geometry with a triangular surface grid and the volume of the cell with a tetrahedral volume grid. In the following we denote the discrete volume of the neuron as Ω and each arising control volume box as *B_i_*, *i* = 1, …, *n*. Therefore Ω = ∪^*n*^_1_*B_i_*. The Finite Volume method (cf. Eymard et al., 2000) defines an ansatz space *V_h_* ⊂ *H*^1^(Ω) which is spanned by piecewise linear ansatz functions Λ_*i*_($\vec{x}$_*i*_) = δ_*ij*_ and is used as the approximation for the solution *u*. The approximation of *u* is then defined as the linear combination of these ansatz functions, resulting in a system of linear equations

(32) $$\sum_{j}^{n}A_{ij}u_{j}=b_{i}$$

where *A* is the stiffness matrix. To solve the linear system of equations, we applied a geometric multigrid solver (smoother ILU) as a preconditioner along with BiCGstab as the base solver for the linear part (inner loop) and a Newton solver for the non-linear part (outer loop), see e.g., (Hackbusch, 1985, 2003). The numerical setup and simulations were carried out in uG [see Bastian et al. (1997); Vogel et al. (in press)].

### Conflict of interest statement

The authors declare that the research was conducted in the absence of any commercial or financial relationships that could be construed as a potential conflict of interest.

## Supplementary material

Supplemental Movie 1 **Simulation of 200 ms real time, perspective 1**.

Supplemental Movie 2 **Simulation of 200 ms real time, perspective 2**.
