# Supplementary material for: 1D-3D hybrid modeling—from multi-compartment models to full resolution models in space and time
Source: Front Neuroinform. 2014 Jul 29;8:68. doi: 10.3389/fninf.2014.00068 (PMC4114301; doi:10.3389/fninf.2014.00068)
Supplement: Supplementary file 3 [file Presentation1.PDF]

SUPPLEMENTAL DATA

A

```
create soma // create soma
ndend = 3 // 3 dendrites
create dend[ndend]// create dendrites

// define soma's membrane parameteters
// and morphology (diameter, length, resisitivity)
soma diam=18.8
soma L = 18.8
soma Ra=123.0

// insert electrical mechanism
// here use hoxkin-huxley for soma
insert hh
gnabar_hh= 0.2
gl_hh = .0001666
el_hh = -60.0
insert pas

// define dendrites parameters (cf above)
dend[0] { nseg = 5 diam = 3.18 L = 701.9 Ra = 123}
dend[1] { nseg = 5 diam = 2.0 L = 549.1 Ra = 123 }
dend[0] { nseg = 5 diam = 2.1 L = 637.4 Ra = 123 }
for i = 0, ndend-1 dend[i] { insert pas g_pas = .0001667 e_pas = -60.0 }

// interconnect compartments
connect dend[0](0), soma(0)
connect dend[1](0), soma(1)
connect dend[2](0), soma(2)
```

B

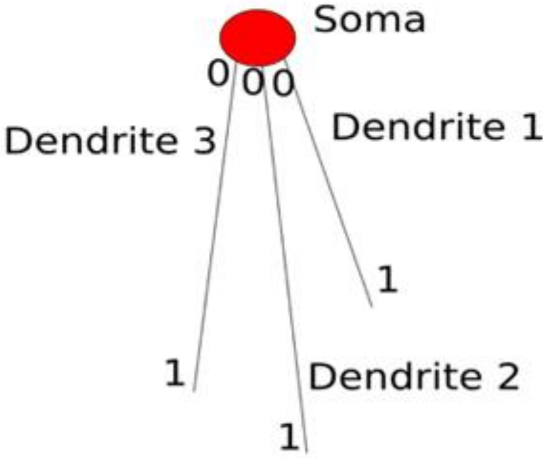

Supplemental Figure S1. Geometry specification by means of the NEURON hoc file format. For demonstration purposes a rather simple geometry is specified, i. e. three dendrites and a soma are defined and each dendrite is connected to the soma by attaching the beginning of each dendrite to the soma. A: The hoc-script code for describing the simple geometry, note that the indices 0 and 1 in parentheses in this case indicate the start respectively the end of each dendrite. B: Visualization of the geometry which was designed by the hoc-script in A.

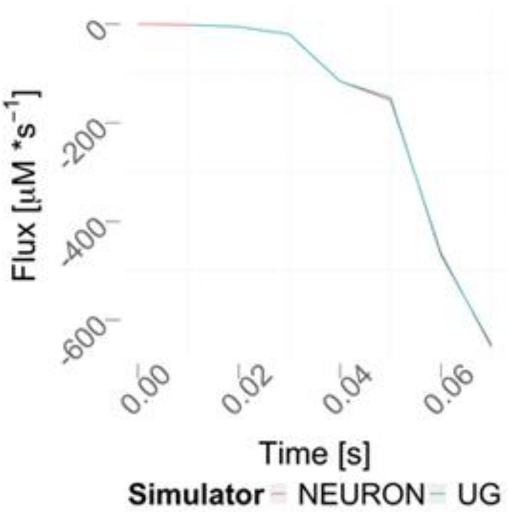

Supplemental Figure S2. Comparison of the VGCC-fluxes at specific points over time, computed in uG and in NEURON (using the pre-computed calcium concentrations as documented in the Results), which show good agreement.

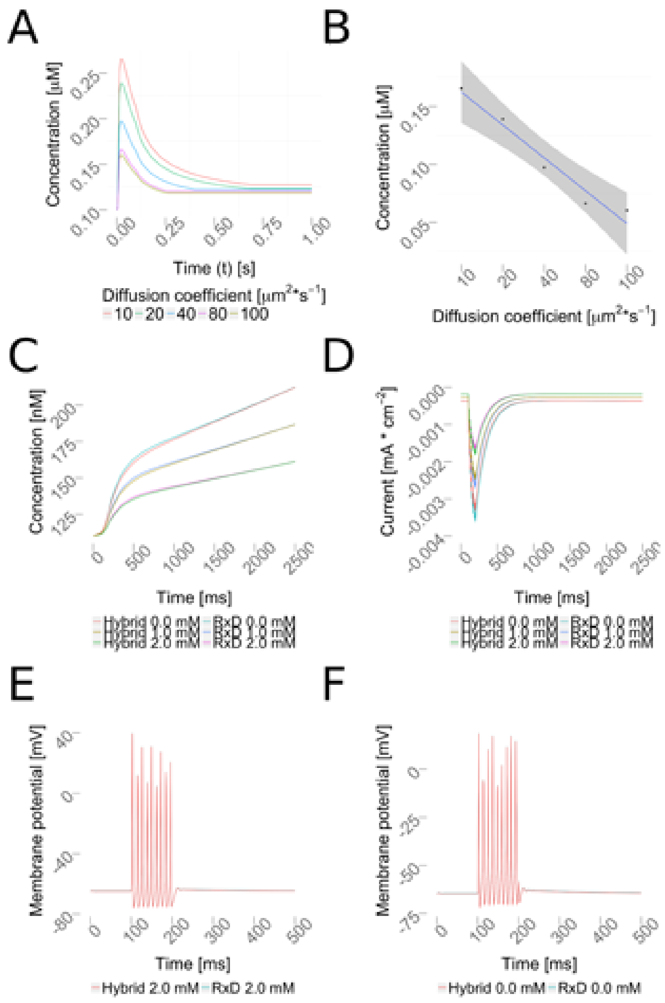

Supplemental Figure S3. Validation of calcium profiles shown in Fig. 7 A using the 1d approximation with the RxD module of NEURON, **McDougal et al.** (2013a). Comparing A and B to Fig. 7 A, B shows good agreement between 1d approximation and 3d model when the intracellular space is assumed homogeneous without intra-cellular obstacles or organelles. C-F: Validation of the hybrid framework on a simplified test case. We used a single dendrite 700 μm length and 3 μm diameter and uniformly distributed N-type calcium channels across the membrane. Intracellular dynamics were reduced to a pure diffusion process in order to allow direct comparison. Since no calcium pumps were inserted in the membrane, calcium concentrations do not return to baseline. The driving force of the calcium channels was varied by changing the extracellular calcium concentration in the Goldman-Hodgkin-Katz model for N-type calcium channels (see Methods). The hybrid framework was compared to simulations run with the RxD module of NEURON, by running a simulation where we injected a 100 nA current at the center of the dendrite for 100 ms, starting at 100 ms simulation time. C: Comparison of the calcium concentrations in the hybrid vs. RxD simulations at three different extracellular calcium concentrations (0.0 mM, 1.0 mM, 2.0 mM), D: Comparison of the calcium currents in the hybrid vs. RxD simulations at three different extracellular concentrations. C and D show overall good agreement between the two approaches for the simplified test case. E, F: Shown are the corresponding membrane potential traces at 2.0 mM and 0.0 mM extracellular calcium, for the hybrid and RxD simulations. Note, that the agreement between the two models are such, that the RxD traces are ideally overlapping with the hybrid traces.
